# Navigating Interdependence: The End-of-Life Caregiving Process of Adult Only Children Caring for Their Parent

**DOI:** 10.1093/geroni/igaf122.2616

**Published:** 2025-12-31

**Authors:** Zhiqi Yi, Nili Wang, Sarah Jen, Shuo Xu

**Affiliations:** University of Kansas, Lawrence, Kansas, United States; Beijing Normal University, Beijing, Beijing, China (People’s Republic); University of Kansas, Lawrence, Kansas, United States; Renmin University of China, Beijing, Beijing, China (People’s Republic)

## Abstract

The one-child policy has shaped the life experiences of millions of only children in China and providing end-of-life care to their aging parents is becoming a critical social issue. Their unique lived experiences likely lead to distinctive end-of-life caregiving experiences, however, to date no existing literature has investigated this phenomenon. This study uses a convenience sample of 15 adult only children who provided at least one month of end-of-life care leading up to their parents’ death to explore their end-of-life caregiving process. Adopting a social constructionist paradigm, this study uses the “In-Vivo” approach to theory-building and thematic analysis to visualize and present findings. The emergent caregiving process encompasses four phases of interdependence depicted through the metaphor of charting a sailing voyage: 1) Sailing Out of the Harbor: Continued Interdependence; 2) Navigating Back to the Harbor: Inverted Interdependence and Shifting Life Emphasis; 3) The Collapsing Harbor: Decoupling Interdependence and Preparation for Bereaved Life; 4) The Navigator’s and Harbor’s Reconstruction: Divergent Interdependence and Social Life. Three turning points sequentially connect the four phases: 1) realizing the severity of the illness and caregiving should be the main task; 2) sensing the possible coming death; 3) death of or farewell to the parents. The parent-child relationship anchors the process, while sociocultural context and illness progression drive the process forward. Practitioners can provide competent psychosocial care in response to the unique dynamics of interdependence and caregiving stress in individual cases. Advocacy for death education and policy to promote hospice and palliative care are recommended.

